# *Mycobacterium xenopi* infection of the kidney and lymph nodes: A case report

**DOI:** 10.1515/med-2023-0646

**Published:** 2023-02-07

**Authors:** Guoyang Zeng, Jiajie Lu

**Affiliations:** Infectious Disease Department, Shifang People’s Hospital, Shifang 618400, Sichuan, China; Infectious Disease Department, The Center of Infectious Diseases, West China Hospital, Sichuan University, Chengdu 610041, Sichuan, China

**Keywords:** combination treatment, metagenomic next-generation sequencing, nontuberculous mycobacteria, extrapulmonary infection, fever

## Abstract

The incidence of nontuberculous mycobacterial (NTM) infection has been increasing globally. Further, it has been reported that early NTM infection diagnosis and treatment can considerably improve patient prognosis. However, traditional methods for detecting pathogenic microorganisms are associated with several limitations, and optimal treatment regimens for several NTM infections have not yet been established. Here, we report the case of a 22-year-old woman with renal and lymph node *Mycobacterium xenopi* infection. This patient presented with repeated fever and systemic lymphadenopathy events for more than 2 years, but the etiology of the disease was unclear. We performed metagenomic next-generation sequencing (mNGS) using tissue sections from the patient’s left kidney and successfully identified *M. xenopi*. Thereafter, the patient’s condition was effectively controlled via treatment with rifampicin, clarithromycin, and ethambutol hydrochloride (orally administered after hemodialysis). Further, this case showed that the clinical symptoms of NTM infection are atypical and highly occult, especially for extrapulmonary NTM infections, which are difficult to diagnose. Therefore, mNGS may be a powerful tool for diagnosing NTM infections. The combination therapy used showed efficacy and thus could serve as a reference treatment for kidney and lymph node *M. xenopi* infection.

## 1 Introduction

Nontuberculous mycobacteria (NTM) refer to all mycobacteria, excluding *Mycobacterium tuberculosis* complex and *Mycobacterium leprae*. To date, more than 190 species and 14 subspecies of NTM, which mainly invade the human body via the respiratory tract, gastrointestinal tract, and skin, with water and soil as primary transmission routes, have been identified [1]. Pulmonary infection is the most common manifestation of NTM, accounting for approximately 90% of infections. Other common NTM infections include lymph node, bone, skin, and soft tissue infections as well as disseminated infections [2,3]. Further, NTM are conditional pathogens, and the pathogenesis of their infections is similar to that of tuberculosis [4]. Here, we report a case of a 22-year-old woman with renal and lymph node *M. xenopi* infection. Our literature search did not reveal any relevant case reports.

## 2 Case presentation

**Figure 1:**
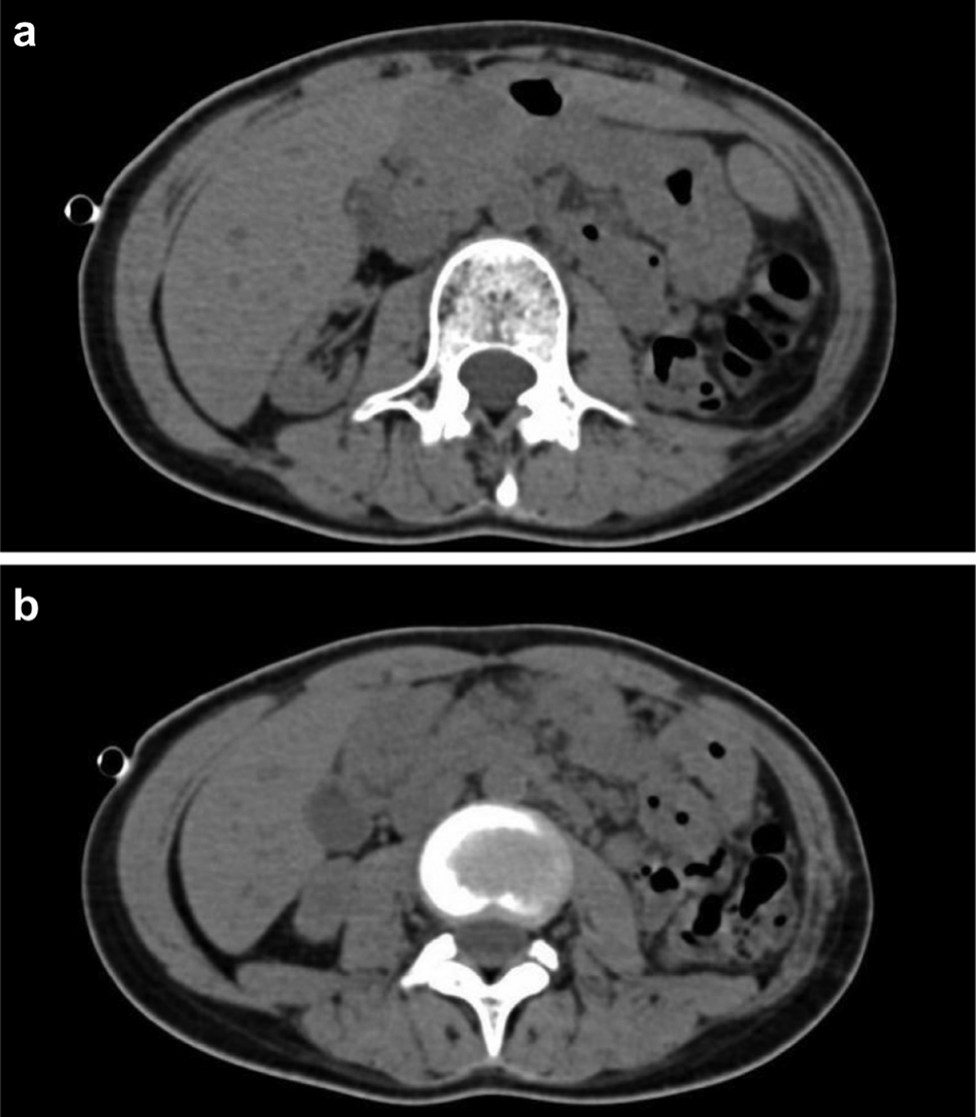
Abdominal computerized tomography images showing (a) left renal agenesis and (b) right renal atrophy.

**Figure 2:**
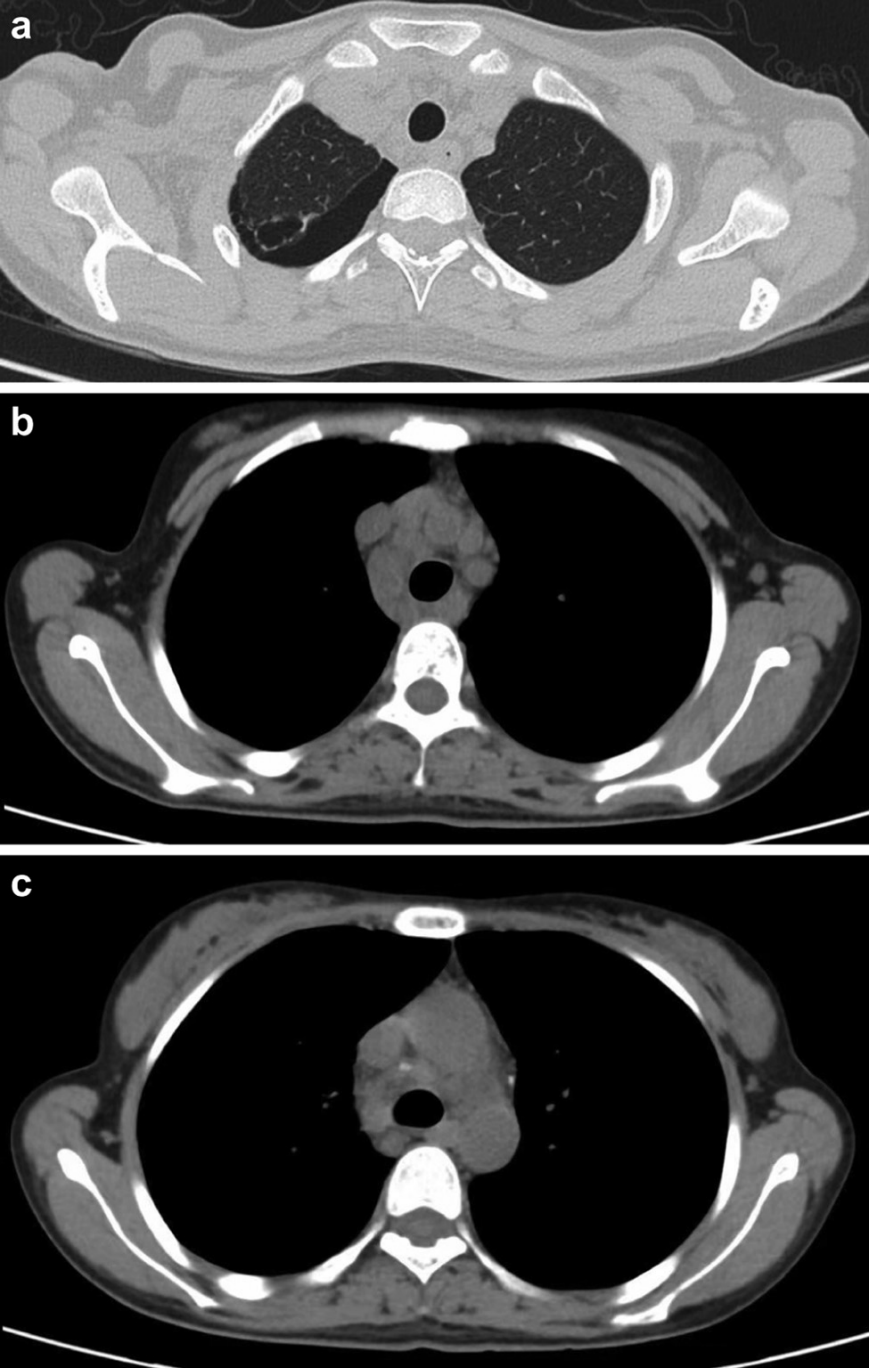
Chest computerized tomography images showing (a) right pneumothorax, (b) lymphadenectasis in the left axilla, and (c) lymphadenectasis in the mediastinum.

**Figure 3:**
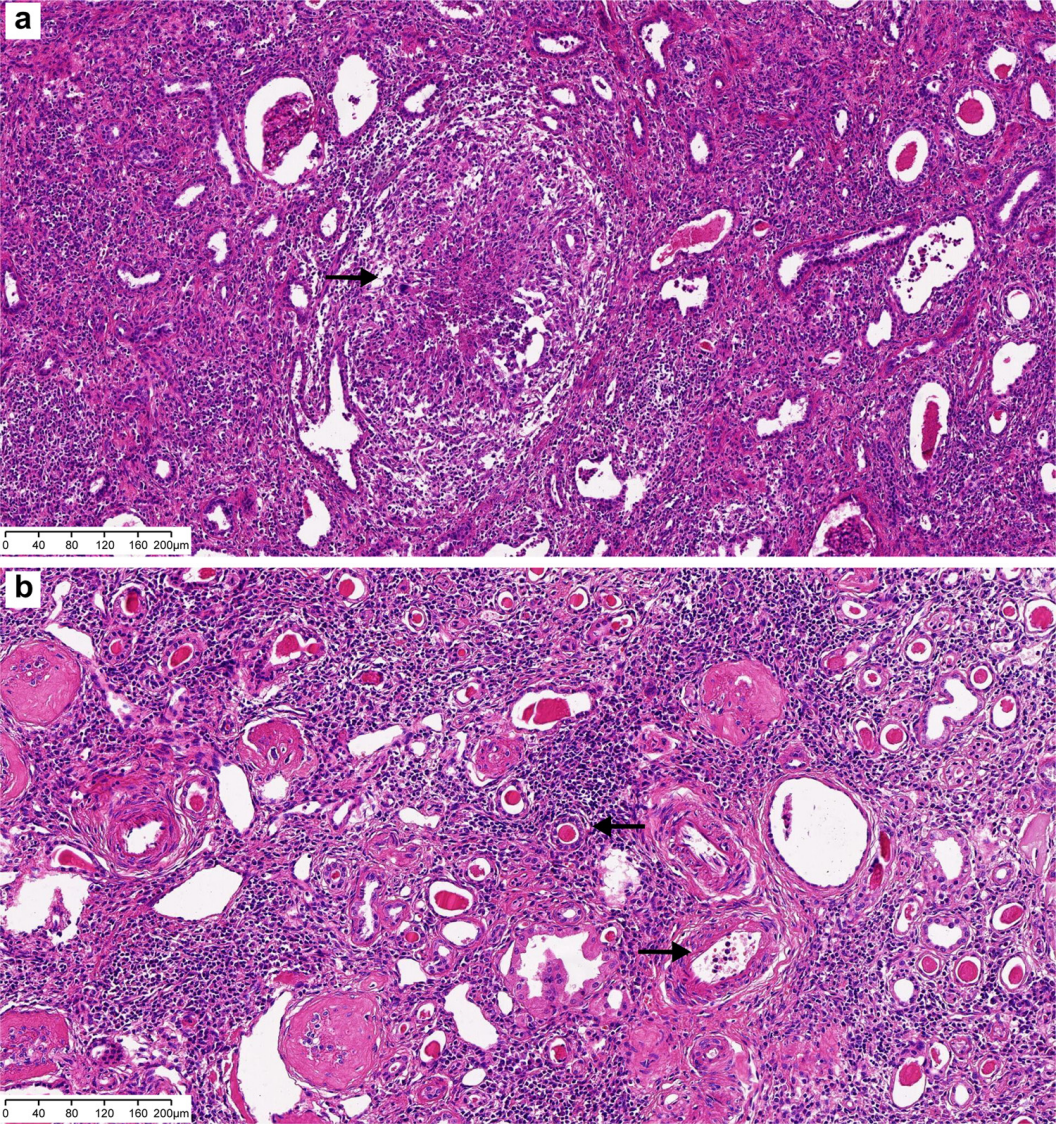
Left kidney tissue sections from the patient showing (pathological HE staining scale bar: 200 µm): (a) noncaseating granulomas (indicated using arrows) and (b) biopsy-based infiltration of mixed inflammatory cells and thickening of the walls of small renal blood vessels (indicated using arrows).

A 22-year-old female patient was admitted to West China Hospital of Sichuan University on August 16, 2021, for chronic intermittent fever (temperature ranging from 38 to 39°C) and systemic enlargement of the lymph nodes with no obvious cause; these symptoms had occurred for over a 2-year period. In addition, the patient suffered from chills, dizziness, headache, fatigue, muscle aches, and numbness in all four extremities. The patient had visited other hospitals multiple times, but the cause of the fever and systemic enlargement of the lymph nodes was not successfully identified. This patient was administered cefoperazone sodium and sulbactam sodium via injection as first-line antimicrobial therapy; however, this treatment showed poor therapeutic efficacy. Further, the patient was later admitted to a local hospital for gradual renal impairment and was diagnosed with uremia, Sjögren’s syndrome, bilateral renal atrophy, and antineutrophil cytoplasmic antibody (ANCA)-associated vasculitis. She subsequently underwent a left nephrectomy. However, after surgery, she still experienced recurrent fever and systemic enlargement of the lymph nodes. She then visited our hospital for a clear diagnosis and further treatment. She was conscious on admission, with a body temperature of 37.3°C, and displayed no rashes or subcutaneous hemorrhage. A firm, mobile, and nontender lymph node of approximately 2 cm in diameter, with no adherence to the surrounding tissues, was palpable in the right supraclavicular area. Local redness and swelling of the skin were not observed. Routine blood examination revealed the following: red blood cell count, 2.66 × 10^12^/L; hemoglobin level, 81 g/L; packed cell volume, 0.26 L/L; white blood cell count, 5.47 × 10^9^/L; platelet count, 147 × 10^9^/L; and ferritin level, 1042.00 ng/mL. Renal function tests revealed the following: urea level, 10.8 mmol/L; creatinine level, 578 µmol/L; estimated glomerular filtration rate, 8.28 mL/min/1.73 m^2^; and procalcitonin level, 0.97 ng/mL. Further, immunological examination revealed the following: absolute CD4 count, 469 cells/µL; absolute CD3 count, 994 cells/µL; absolute CD8 count, 424 cells/µL; immunoglobulin G level, 29.40 g/L; immunoglobulin A level, 5910.00 mg/L; complement C3 level, 0.7120 g/L; rheumatoid factor level, 137.00 IU/mL; antinuclear antibody level, + 1:1,000 granular cytoplasm; anti-SS-A antibody, ++; and anti-Ro-52 antibody, ++. Sputum tested using the GeneXpert system (Cepheid, Sunnyvale, CA, USA) was negative for *M. tuberculosis* infection although the interferon-gamma release assay showed a positive result (89.49 pg/mL). In addition, a color Doppler ultrasound of the lymph nodes showed structurally abnormal and enlarged lymph nodes in the right supraclavicular area and left neck root area. Further, a color Doppler ultrasound of the urinary system revealed left renal agenesis, right renal atrophy, and the presence of parenchymal lesions in the right kidney. Abdominal computerized tomography (CT) imaging confirmed left renal agenesis and right renal atrophy (Figure 1). The CT imaging of the chest showed pulmonary bullae on the apex of the right lung and right pneumothorax, with approximately 15% compression of the right lung. An increased number of enlarged lymph nodes were also observed in the left axilla and mediastinum (Figure 2). We also subjected a kidney tissue sample from the patient (obtained from the previous hospitalization) to pathological examination at our hospital. The histological results showed the infiltration of a large number of mixed inflammatory cells into the renal interstitium, the presence of noncaseating granulomas in the focal areas, and thickening of the walls of the glomerular capsules as well as small renal blood vessels (Figure 3).

On August 24, 2021, this left kidney section was further subjected to metagenomic next-generation sequencing (mNGS) for pathogen identification. The sample was first subjected to sample enrichment via centrifugation at 15,000 *g* for 10 min at 4°C. Thereafter, microbial DNA was extracted from 200 µL of the enriched sample and purified via the magnetic bead method (Magen Biotechnology, Guangzhou, China). The extracted DNA was then used to construct a metagenomic library (library size: ∼300 bp) according to the instructions of the Nextera XT library building kit (Illumina, San Diego, CA, USA). Next, the library size was analyzed using the 2100 Bioanalyzer (Agilent, Santa Clara, CA, USA) and quantified via qPCR (Bio-Rad CFX96, Hercules, CA, USA). After mixing the library with different index adapter sequences in equal amounts, high-throughput sequencing was performed using the Illumina NextSeq 550DX sequencing platform (San Diego, CA, USA; sequencing strategy: SE75). The data obtained were filtered using FastQC software, and valid sequences (clean reads) were obtained after removing joints, low-quality bases, and very short sequences. Bowtie2 was used to align the clean reads with the human genome reference sequence (version GRCh38), to remove host-related reads, and for optimization. The test showed the presence of *M. xenopi* (15 specific reads), *Pseudomonas aeruginosa* (27 specific reads), *Bacteroides fragilis* (18 specific reads), *Streptococcus pneumoniae* (13 specific reads), *Fusobacterium mortiferum* (9 specific reads), and *Lodderomyces elongisporus* (9 specific reads) (Table 1).

**Table 1:** Pathogens detected in tissue sections of the patient’s left kidney using metagenomic next-generation sequencing

List of bacteria				
Gram stain test	Genus	Species	Detected reads, *n* (%)	Confidence level (%)
B:G−	*Pseudomonas*	*Pseudomonas aeruginosa*	27 (32.93)	99
B:G−	*Bacteroides*	*Bacteroides fragilis*	18 (21.95)	99
B:G+	*Streptococcus*	*Streptococcus pneumoniae*	13 (15.85)	99
B:G−	*Fusobacterium*	*Fusobacterium mortiferums*	9 (10.98)	99

List of fungi				
Genus	Species	Detected reads, *n* (%)	Confidence level (%)	
*Lodderomyces*	*Lodderomyces elongisporus*	9 (100)	99	

List of special pathogens				
The detected mycobacterium complex				
Name	Detected reads, *n* (%)	Identified confidence level (%)		
*Mycobacterium xenopi*	15 (18.29)	99		

After hospitalization, the patient’s body temperature fluctuated between 37.0°C and 39.5°C. A routine blood examination was repeated on September 5, 2021, which revealed a red blood cell count of 2.54 × 10^12^/L, hemoglobin level of 78 g/L, and packed cell volume of 0.25 L/L. Pharmacological treatment with 450 mg rifampicin once a day, 250 mg clarithromycin twice a day, and 0.75 g ethambutol hydrochloride three times a week (oral administration after hemodialysis) was initiated on September 7, 2021. After 3 days, the patient’s body temperature returned to the normal range. During the 4-month follow-up period, her body temperature remained within the normal range, and a color Doppler ultrasound of lymph nodes in the neck revealed significant decreases in the sizes of the cervical lymph nodes.


**Ethical approval:** The study was performed in compliance with all relevant national regulations and institutional policies and in accordance with the tenets of the Declaration of Helsinki. The Institutional Review Board of West China Hospital of Sichuan University approved the study protocol (approval document of biomedical ethics review committee of West China Hospital of Sichuan University, 2022 review No. 662).
**Informed consent:** Written informed consent for publication was obtained from the patient.

## 3 Discussion

*M. xenopi*, which is a nonchromogenic Group III *Mycobacterium* that is commonly encountered along the Pacific coastline, was first isolated from the skin of a toad in 1959 [5]. Further, it is frequently detected in water, soil, tap water supply systems, and shower nozzles. It mainly causes lung lesions, but can also affect the skin, soft tissues, bones, joints, and bone marrow [1,6,7,8,9]. It is similar to *M. tuberculosis* in terms of etiological characteristics and can also produce a positive result in the acid-fast stain test. Therefore, further bacteriological identification is needed to distinguish it from *M. tuberculosis*. The key to diagnosing NTM infection is to isolate and culture the pathogen; however, traditional methods for isolating and culturing pathogenic microorganisms have low positive rates and long detection times. In addition, with their use, it is difficult to accurately identify strains, and this may lead to misdiagnosis. Our patient complained of chronic recurrent fever, lymph node enlargement, and renal impairment with insidious onset, long disease duration, and no specificity. A variety of routine medical examinations had been performed, but the cause for these symptoms was not determined. To confirm the pathogen, we initially planned to perform a cervical lymph node or renal biopsy for the associated pathological and etiological examinations. However, the patient had a pneumothorax, which made the procedure risky. Furthermore, the patient and her family members refused permission for tissue biopsy. Therefore, we performed mNGS using the patient’s renal tissue sections obtained from other hospitals where she underwent treatment. Multiple bacterial species, including *M. xenopi, P. aeruginosa, B. fragilis*, *S. pneumoniae, F. mortiferum*, and *L. elongisporus*, were detected.

Regarding the mNGS test results, we considered that cefoperazone sodium and sulbactam sodium could be useful for the patient. Specifically, the antibacterial spectrum of this drug covers *P. aeruginosa*, implying that if the infection was caused by *P. aeruginosa*, then her condition would have been kept under control. However, given that her condition progressively worsened, it was therefore unlikely that *P. aeruginosa* was responsible for the long-term recurrence of fever, swollen lymph nodes, and even kidney failure in the patient. Further, it was evident that the traditional cephalosporin antibiotic treatment was not effective against the *M. xenopi* infection, considering the patient’s 2-year medical history and the symptoms of NTM infection. Therefore, we considered that administering treatment for *M. xenopi* infection was a viable option. Thus, the patient’s condition improved considerably, confirming our diagnosis of NTM infection based on the mNGS results.

Owing to the current trend of pathogen diversification, conventional microbiological tests, including microscopic analysis, culture assays, nucleic acid amplification tests, and immunological assays, are associated with several limitations with respect to providing a reliable diagnosis for unknown pathogenic microorganisms [10,11]. However, mNGS technology enables the unbiased sequencing of all nucleic acids in a sample and offers the possibility to detect the sequences of suspected pathogens via the utilization of algorithms based on large databases of pathogenic microorganisms. Further, it does not rely on traditional microbial culture, does not require specific primers, has a short detection time, achieves accurate detection, and has a high strain identification ability. Therefore, we believe that mNGS is an important method for detecting NTM infection.

Currently, drugs such as rifabutin, macrolides, moxifloxacin, linezolid, isoniazid, rifampicin, ethambutol, and ciprofloxacin are used to treat *M. xenopi* infection [1,6,12,13,14,15]. Notably, *in vitro* drug sensitivity assays have indicated that most *M. xenopi* variants are sensitive to rifabutin, macrolides, moxifloxacin, and linezolid, but only moderately sensitive to isoniazid, rifampicin, ethambutol, and ciprofloxacin [1,6,12,13,14,15]. Among these drugs, clarithromycin has a strong antibacterial effect on several NTM, including *Mycobacterium avium* complex, *M. xenopi*, *Mycobacterium gordonae*, *Mycobacterium kansasii*, *Mycobacterium fortuitum*, and *Mycobacterium abscessus* [1,6,12,13]. The minimum inhibitory concentration of rifampicin alone is considerably higher than that observed when it is used in combination with ethambutol [16]. The 2017 guidelines of the British Thoracic Society for the management of NTM pulmonary disease (PD) recommends the use of a four-drug combination, comprising rifampicin, ethambutol, macrolide, and quinolones (or isoniazid) for the treatment of *M. xenopi*-PD in case of infection [6]. Further, regarding the treatment of NTM PD, the 2020 guidelines of the American Thoracic Society recommend a regimen comprising at least three drugs, i.e., rifampicin, ethambutol, macrolides, and/or fluoroquinolones (e.g., moxifloxacin) as treatment for *M. xenopi*-PD [1]. In addition, both guidelines provide corresponding recommendations regarding NTM-PD treatment duration, e.g., for the treatment of *MAC*-PD, *M. kansasii*-PD, *M. xenopi*-PD, and *M. abscessus*-PD using antibiotics, and the treatment should continue for a minimum of 12 months after culture conversion [1,6]. However, relevant literature on the treatment time and medication standard for extrapulmonary infection NTM is limited. On the basis of recommendations for *M. xenopi*-PD treatment provided by these two guidelines, we developed a treatment strategy for our patient with kidney and lymph node *M. xenopi* infection. The patient showed peripheral neuropathy, ANCA-associated vasculitis, and moderate anemia (78 g/L) before therapy initiation. Thus, moxifloxacin or linezolid was not added to the treatment regimen for safety reasons; we opted for a combination of rifampicin, clarithromycin, and ethambutol. The recommended dosage of these drugs in the aforementioned guidelines are as follows: clarithromycin, 500 mg twice per day (renal impairment, e.g., CrCl < 30 mL/min, does is reduced by 50%); rifampicin, 10 mg/kg (450 mg or 600 mg) per day; ethambutol, 15 mg/kg per day (renal impairment, dosing interval is increased, e.g., 15–25 mg/kg three times per week) [1,6]. Considering the patient’s condition, we used 450 mg rifampicin once a day, 250 mg clarithromycin twice a day, and 0.75 g ethambutol hydrochloride three times a week (oral administration after hemodialysis). After treatment, the body temperature of the patient returned to normal (axillary temperature, 36–37°C) and remained within the normal range during the 4-month follow-up period. Further, a color Doppler ultrasound showed that the previously swollen cervical lymph nodes significantly decreased in size. The follow-up results also confirmed the safety and efficacy of the therapeutic regimen. However, it has been observed that the recurrence rate of infection after NTM treatment is high. Some studies have shown that the recurrence rate of *MAC*-PD is as high as 8.3–48% [17]. Specifically, in one previous study, it was observed that of 13 patients with lymph node infection with NTM, 11 patients experienced recurrent infection by the same microorganism 2–7 years after treatment discontinuation [18]. Long-term treatment may prevent recurrence; thus, we believe that the treatment course for this case should be 1 year or more.

NTM can cause infection in individuals with normal immune function, while individuals with immunodeficiency, such as those with HIV infection, AIDS, autoimmune diseases, and cancer, are at a higher risk of infection [19,20,21,22]. It has also been observed that individuals with basic lung diseases are prone to suffer from NTM PD. Further, gastroesophageal reflux, rheumatoid arthritis, vitamin D deficiency, the use of immunosuppressive drugs, and malnutrition have been identified as risk factors for NTM disease [23,24]. Combined with the patient’s immune-related examination, we considered that the patient may have autoimmune diseases, such as Sjogren’s syndrome and ANCA-related vasculitis, and the decrease in the CD4 count of the patient further confirmed a low immune function. Therefore, we believe that NTM infection is associated with impaired immune function.

## 4 Conclusions

Currently, treating NTM infection is still challenging. Further, optimal treatment schemes for many NTM infections have not yet been established; some recommended drug treatment schemes fail to achieve satisfactory results. Thus, further clinical trials are needed to provide a theoretical and practical basis for efficient NTM infection treatment [15,16]. This case confirmed that the three-drug combination treatment regimen, comprising rifampicin, clarithromycin, and ethambutol hydrochloride, is safe and effective as the treatment for kidney and lymph node *M. xenopi* infection. In addition, the study suggests that mNGS is an important and efficient method for detecting NTM infection and should be performed as early as possible.
